# Predictive Value of the Systemic Immune-Inflammation Index for Intravenous Immunoglobulin Resistance and Cardiovascular Complications in Kawasaki Disease

**DOI:** 10.3389/fcvm.2021.711007

**Published:** 2021-08-24

**Authors:** Xiaoliang Liu, Shuran Shao, Lin Wang, Nanjun Zhang, Mei Wu, Lei Liu, Li Zhao, Yimin Hua, Kaiyu Zhou, Chunyan Luo, Yuxin Deng, Chuan Wang

**Affiliations:** ^1^Department of Pediatric Cardiology, West China Second University Hospital, Sichuan University, Chengdu, China; ^2^Key Laboratory of Birth Defects and Related Diseases of Women and Children (Sichuan University), Ministry of Education, Chengdu, China; ^3^Key Laboratory of Development and Diseases of Women and Children of Sichuan Province, West China Second University Hospital, Sichuan University, Chengdu, China; ^4^West China Medical School of Sichuan University, Chengdu, China; ^5^Longquanyi District of Chengdu Maternity & Child Health Care Hospital, Chengdu, China; ^6^Department of Pediatrics, West China Second University Hospital, Sichuan University, Chengdu, China; ^7^The Cardiac Development and Early Intervention Unit, West China Institute of Women and Children's Health, West China Second University Hospital, Sichuan University, Chengdu, China; ^8^Huaxi MR Research Center (HMRRC), Department of Radiology, West China Hospital, Sichuan University, Chengdu, China; ^9^Department of Biology, College of Science and Technology, Wenzhou-Kean University, Wenzhou, China

**Keywords:** Kawasaki disease, systemic immune-inflammation index, intravenous immunoglobulin resistance, neutrophil-to-lymphocyte ratio, platelet-to-lymphocyte ratio, cardiovascular complications

## Abstract

**Background:** The prediction of intravenous immunoglobulin (IVIG) resistance and cardiovascular complications are critically clinical issues in Kawasaki disease (KD). This prospective study firstly aimed to determine the predictive ability of the systemic immune inflammation index (SII) for IVIG resistance and cardiovascular complications and compare the prognostic accuracy of SII with that of neutrophil to lymphocyte ratio (NLR) and platelet to lymphocyte ratio (PLR).

**Methods:** Patients with KD were divided into different groups according to the presence of IVIG resistance or cardiovascular complications (coronary artery lesions, valve regurgitation, myocarditis, pericardial effusion, and Kawasaki disease shock syndrome [KDSS]). The clinical and laboratory parameters were compared. Further analysis stratified by platelet level was performed. Multivariate logistic regression analysis was used to identify predictors for IVIG resistance and cardiovascular complications. The receiver operating characteristic (ROC) curve was applied to assess and compare the ability of SII, NLR, and PLR for predicting IVIG resistance and cardiovascular complications.

**Results:** SII was significantly higher in KD patients with IVIG-resistance, myocarditis, valve regurgitation, and KDSS. It was identified as an independent risk factor for IVIG resistance, myocarditis, and valve regurgitation. For KD patients with thrombocytopenia, there were no significant differences in SII between KD patients with IVIG resistance/cardiovascular complications and those without. The best cutoff values of SII for IVIG resistance, myocarditis, valve regurgitation, and KDSS prediction in the whole cohort were 1331.4 × 10^9^, 1368.6 × 10^9^, 1002.4 × 10^9^, and 1485.4 × 10^9^, with a corresponding sensitivity of 0.525, 0.614, 0.754, and 0.670, a specificity of 0.711, 0.723, 0.584, and 0.730, respectively. The predictive value of SII for both IVIG resistance and cardiovascular complications were not superior to that of NLR.

**Conclusion:** Although the parameter of SII may predict IVIG resistance, myocarditis, valve regurgitation, and KDSS in KD as a single parameter, its predictive ability was not good enough and not superior to NLR. SII might not be applicable in patients with KD having thrombocytopenia.

## Introduction

Kawasaki disease (KD) is an acute systemic vasculitis with unknown etiology, predominantly affecting infants and children (1). Timely intravenous immunoglobulin (IVIG) treatment is substantially effective; however, approximately 10–20% of patients with KD are resistant to IVIG treatment and substantially develop a risk of coronary artery lesions (CALs) as the most serious cardiac sequela (2). Other cardiovascular sequelae like myocarditis, valvular abnormalities, pericarditis, and KD shock syndrome (KDSS) are now being increasingly recognized (3). Early identification and appropriate treatment of these complications is also of paramount importance and could improve the prognosis of KD patients (4). Therefore, it is substantially necessary for clinicians to get a useful tool for predicting KD patients with IVIG resistance and cardiovascular complications. Patients may benefit from adjunctive therapies for primary treatment, including corticosteroids, infliximab, plasma exchange, cytotoxic agents (1, 5), because the aforementioned clinical concerns are associated with severe inflammation burden. Although several risk-scoring systems have been developed in Japan (6–8), their effectiveness in predicting IVIG resistance and cardiovascular complications varies in different populations (4, 9, 10).

It has been proved that neutrophil, as a classical marker of inflammation, plays a critical role in the initial immune response in the first pathological process of KD. On the other hand, immunosuppression will be triggered by the release of various anti-inflammatory cytokines resulting in the apoptosis of lymphocytes, which participate in the second pathological process of KD (11). Thus, the divergence of the neutrophil (higher) and lymphocyte (lower) counts might indicate that patients are undergoing severe inflammatory reactions and clinical course of KD (12–14). Besides, the platelet count might also elevate due to megakaryocytic proliferation stimulated by proinflammatory cytokines, reflecting the activity of the inflammatory activation (15). However, a single inflammatory parameter may be easily influenced by other factors; therefore, the merging information of inflammatory parameters, namely, neutrophil-to-lymphocyte ratio (NLR), platelet-to-lymphocyte ratio (PLR), maybe theoretically more reliable and have the potentials as powerful candidates to evaluate the inflammatory and immune status. Previous studies (12, 13, 16, 17) and ours (14) have identified NLR and PLR as independent risk factors for IVIG resistance and/or CALs; nonetheless, their predictive abilities were still not good enough (14).

Notably, as a novel systemic inflammatory index merging the information of lymphocyte, neutrophil, and platelet counts, the parameter of systemic immune-inflammation index [SII, platelet count × (neutrophil count/lymphocyte count)] has been recently proposed as a powerful prognostic indicator of poor outcome in patients with cancers (18–20) and coronary artery diseases (21). Prior findings suggested a higher SII was better to reflect the balance of host inflammatory and immune status than NLR and PLR (19, 22, 23); nonetheless, the potential effects of SII have not been elaborated in patients with KD.

Therefore, in the present study, we prospectively investigated the predictive value of SII in predicting IVIG resistance and cardiovascular complications including CALs, myocarditis, valve regurgitation, pericarditis as well as KDSS in KD, and compared the prognostic accuracy of SII with that of neutrophil to lymphocyte ratio (NLR) and platelet to lymphocyte ratio (PLR).

## Patients and Methods

**Figure 1:**
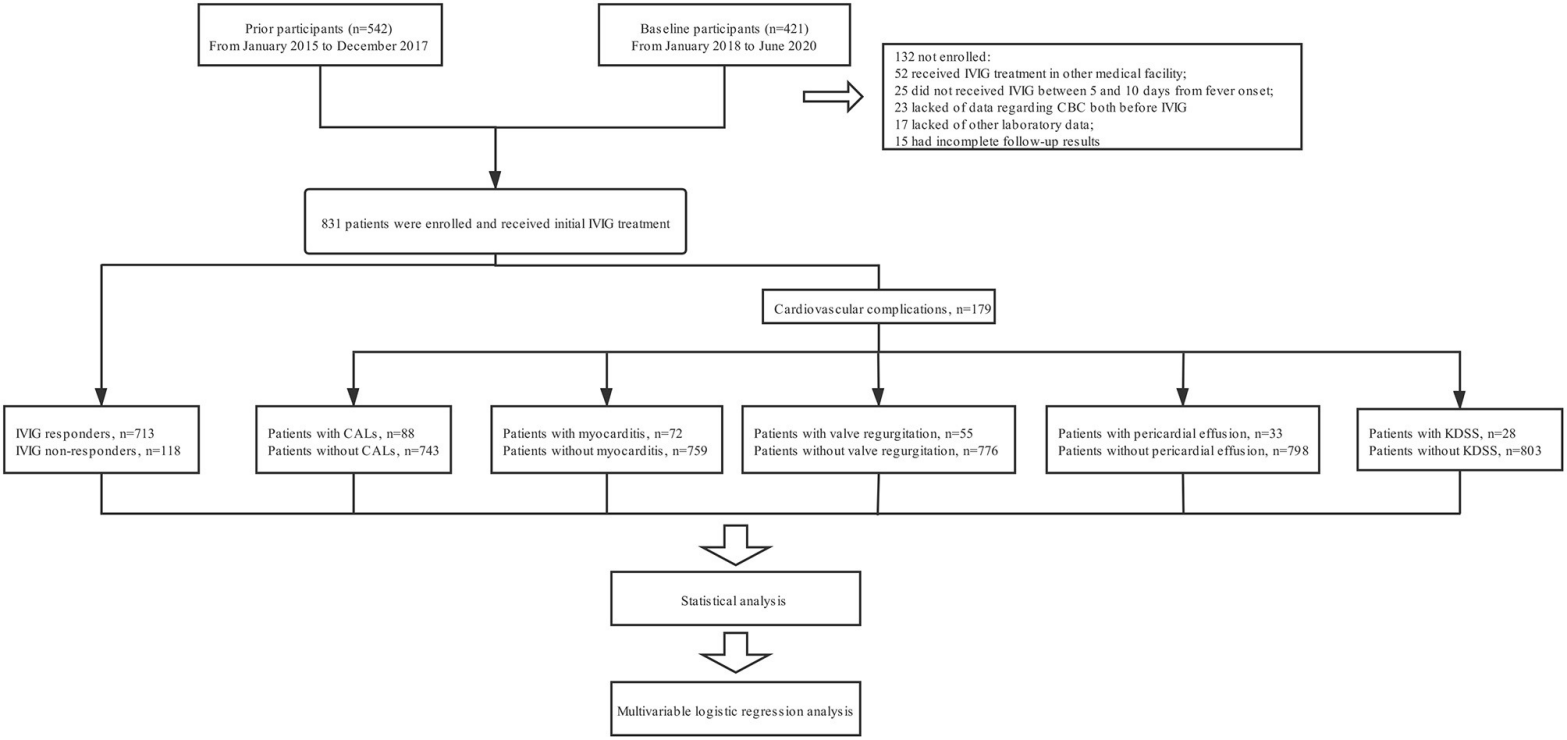
The flowchart of our prospective study. Five hundred forty two patients with KD have been recruited between January 2015 and December 2017 at our hospital. From January 2018 to June 2020, another 421 patients diagnosed with KD were further prospectively enrolled. For this cohort of patients, those who had received IVIG treatment in other medical facilities (*n* = 52) or did not receive IVIG treatment prior to 10 days from fever onset (*n* = 25) were excluded. Additionally, 23 patients were also excluded owing to a lack of data regarding complete blood count (CBC) prior to the initial IVIG. We also excluded 37 patients because other laboratory data (*n* = 17) or follow-up results (*n* = 15) were incomplete. Finally, 831 patients (542 patients recruited between January 2015 and December 2017, 289 patients recruited between January 2018 and June 2020) were enrolled for analysis. All patients with KD were divided into different groups stratified by the presence of IVIG resistance (*n* = 118) and cardiovascular complications (*n* = 179), including CALs (*n* = 88), myocarditis (*n* = 72), valve regurgitation (*n* = 55), pericardial effusion (*n* = 33), and KDSS (*n* = 28). No patients received additional treatment such as infliximab, plasma exchange, and cytotoxic agents.

In our prior prospectively study (14), 542 patients with KD have been recruited between January 2015 and December 2017 at West China Second University Hospital of Sichuan University (WCSUH-SCU). From January 2018 to June 2020, another 421 patients diagnosed with KD were further prospectively enrolled. For this cohort of patients, those who had received IVIG treatment in other medical facilities (*n* = 52) or did not receive IVIG treatment prior to 10 days from fever onset (*n* = 25) were excluded. Additionally, 23 patients were also excluded owing to a lack of data regarding complete blood count (CBC) prior to the initial IVIG. We also excluded 37 patients because other laboratory data (*n* = 17) or follow-up results (*n* = 15) were incomplete. Finally, 831 patients (542 patients recruited between January 2015 and December 2017, 289 patients recruited between January 2018 and June 2020) were enrolled for analysis, including 713 patients with initial IVIG response and 118 patients with initial IVIG resistance. All patients with KD were divided into different groups stratified by the presence of IVIG resistance (*n* = 118) and cardiovascular complications (*n* = 179), including CALs (*n* = 88), myocarditis (*n* = 72), valve regurgitation (*n* = 55), pericardial effusion (*n* = 33), and KDSS (*n* = 28) (Figure 1). No patients received additional treatment such as infliximab, plasma exchange, and cytotoxic agents.

The SII was calculated by N × P/L before the initial IVIG infusion (20). NLR and PLR were calculated by N/L and P/L, respectively (13). KD was diagnosed depending on the American Heart Association (AHA) scientific statement on KD (24). KDSS was proposed when patients with KD presented with systolic hypotension for age, a sustained decreasing systolic blood pressure from baseline of ≥20%, or clinical signs of poor perfusion (25). The diagnosis of pericardial effusion required more than 1 mm of fluid (26). Patients with KD who present elevated levels of cardiac troponin I (cTnI, >0.06 ug/mL), and/or cardiac enlargement (z score of at least one cardiac chamber >2.0) (27, 28), and/or left ventricular dysfunction (left ventricular ejection fraction[LVEF] ≤ 55%) (28), and/or arrhythmia, were considered as myocarditis (29). The definition of valve regurgitation was based on the findings of echocardiography (30, 31). CALs were defined on the normalization of dimensions for body surface area (BSA) as *z* scores, according to the AHA scientific statement of KD (24). Data of the basic demographic information, clinical manifestations, hematological examination results, treatment, and follow-up outcomes were collected with standardized questionnaires by two well-trained doctors. Informed written consent was obtained from parents after the nature of this study had been fully explained to them. The study was approved by the University Ethics Committee on Human Subjects at Sichuan University. All research was performed in accordance with relevant guidelines and regulations.

All patients received the same standard treatment regimen of KD. Aspirin (30–50 mg/kg/day) and IVIG (2 g/kg given as a single intravenous infusion) were administered within the first 10 days of illness from fever onset. After patients defervesce for 48–72 h, a tapered dose of aspirin (3–5 mg/kg/day) was administered for 6–8 weeks. If patients had CALs, aspirin was continued until there was no evidence of CALs. If the patient had initial IVIG resistance, which was defined as recurrent or persistent fever or other clinical signs of KD for at least 36 h but not longer than 7 days after initial IVIG, the second IVIG (2 g/kg given as a single intravenous infusion) was administered (1). The fluid resuscitation, vasoactive agents, blood transfusion, and albumin infusion were prescribed individually or in combination in patients with KDSS (4, 32).

## Statistical Analyses

The categorical data were expressed as the number/%. Continuous data were expressed as median with interquartile range (the 25 and 75th percentiles). The normality of the data was determined by the Shapiro-Wilk test and homogeneity test of variance. The comparison between the two groups was performed by the *x*^2^ test, Student's *t*-test, or Mann-Whitney *U* test as appropriate. Numerical variables, which significantly differed between the two groups, were turned into dichotomous variables. Based on the receiver operating characteristic (ROC) curve, cutoff values corresponding to the maximum Youden's index for sensitivity and specificity were selected. According to univariate analysis, variables with statistical significance were subjected to multivariate logistic regression analysis to identify independent risk factors for IVIG resistance and cardiovascular complications. The parameters of N%, L%, PLT, NLR, and PLR were not included in the multivariate logistic regression analysis because of their strong correlations with SII.

ROC analysis was used to determine the discriminating cutoff values of SII, NLR, and PLR in predicting IVIG resistance as well as cardiovascular complications and their corresponding predictive validities. After that, the sensitivity, specificity, positive predictive value (PPV), negative predictive value (NPV), and diagnostic accuracy were assessed. The comparison between ROC curves was performed by the De Long test. A *p*-value <0.05 indicates statistical significance. SPSS 21.0 for Windows (IBM Corporation, Armonk, NY, USA) was used for all the statistical analyses.

## Results

### Predictive Value of SII for IVIG-Resistance in Patients With KD

The parameters of SII (1339.9 [570.5–2299.0] × 10^9^ vs. 848.8 [479.5–1500.7] × 10^9^, *p* < 0.001), as well as NLR (4.57 [2.62–8.45] vs. 2.71 [1.73–4.72], *p* < 0.001) and PLR (132.0 [81.7–222.5] vs. 99.6 [70.0–147.9], *p* < 0.001), were significantly higher in patients with IVIG resistance than patients with IVIG response. After adjusted by age, hemoglobin, CRP, ALT, ALB, TBil, Na^+^, SII was identified as an independent risk factor for initial IVIG resistance. These results are depicted in Table 1.

**Table 1:** Comparison of clinical data between the groups of initial IVIG-response and IVIG-resistance in Kawasaki disease.

	**IVIG-response(*n* = 713)**	**IVIG-resistance(*n* = 118)**	**Univariate analysis *p*-value**	**Multivariate analysis**	
				**OR (95%CI)**	***p*-value**
Male	406 (56.9)	61 (51.7)	0.263		
Age, years	2.1 (1.2–3.6)	2.3 (1.3–4.6)	0.037	1.272 (0.727–2.226)	0.399
**Clinical manifestations**					
Rash	551 (77.3)	98 (83.1)	0.199		
Extremity changes	394 (55.3)	68 (57.6)	0.689		
Conjunctivitis	655 (91.9)	106 (89.8)	0.474		
Oral changes	642 (90.0)	111 (94.1)	0.231		
Cervical lymphadenopathy	318 (44.6)	63 (53.4)	0.093		
Fever duration before initial IVIG, days	5.0 (5.0–6.0)	5.0 (4.0–6.0)	0.212		
Incomplete KD	277 (38.8)	38 (32.2)	0.218		
**Cardiovascular complications**					
CALs	72 (10.1)	16 (13.1)	0.259		
Myocarditis	56 (7.9)	16 (13.6)	0.051		
Valve regurgitation	43 (6.0)	12 (10.2)	0.100		
Pericardial effusion	22 (3.1)	11 (9.3)	0.004		
KDSS	14 (2.0)	14 (11.9)	<0.001		
**Before initial IVIG**					
The time of blood test	5.0 (4.0–6.0)	4.0 (3.0–5.3)	0.705		
WBC, × 10^9^/L	13.3 (10.7–16.6)	13.6 (10.0–16.8)	0.431		
Neutrophil, %	66.2 (56.0–77.0)	76.9 (65.3–84.0)	<0.001		
Lymphocyte, %	24.6 (16.0–33.0)	15.5 (9.9–24.0)	<0.001		
Hemoglobin, g/L	109.0 (102.0–117.0)	106.5 (101.0–114.3)	0.037	1.606 (1.024–2.519)	0.039
PLT, × 10^9^/L	316.0 (257.0–387.0)	287.5 (195.3–346.5)	<0.001		
CRP, mg/L	72.0 (43.0–107.0)	93.6 (62.0–144.3)	<0.001	1.325 (0.773–2.272)	0.306
ESR, mm/h	64.0 (46.0–81.0)	67.0 (48.0–87.0)	0.202		
AST, U/L	33.0 (25.0–49.0)	37.0 (25.0–73.0)	0.120		
ALT, U/L	36.0 (20.0–77.3)	54.0 (27.0–125.0)	0.005	1.155 (0.719–1.855)	0.551
ALB, g/L	38.0 (35.0–41.0)	36.0 (30.3–39.0)	<0.001	2.175 (1.343–3.522)	0.002
TBil, μmol/L	6.0 (4.0–8.5)	7.0 (5.0–19.0)	<0.001	3.382 (1.838–6.226)	<0.001
Na^+^, mmol/L	137.0 (135.0–139.0)	135.0 (133.0–137.0)	<0.001	2.262 (1.442–3.548)	<0.001
NLR	2.71 (1.73–4.72)	4.57(2.62-8.45)	<0.001		
PLR	99.6 (70.0–147.9)	132.0 (81.7–222.5)	<0.001		
SII	848.8 (479.5–1500.7)	1339.9 (570.5–2299.0)	<0.001	1.627 (1.018–2.601)	0.042

*ALB, Albumin; AST, aspartate aminotransferase; ALT, alanine aminotransferase; CRP, C-reactive protein; CALs, coronary artery lesions; ESR, erythrocyte sedimentation rate; IVIG, intravenous immunoglobulin; KD, Kawasaki disease; KDSS, Kawasaki disease shock syndrome; NLR, neutrophil-lymphocyte ratio; PLR, platelet-lymphocyte ratio; TBil, total bilirubin; Na+, sodium; WBC, white blood cell; SII, systemic inflammatory index*.

*The data are presented as the median with the 25 and 75th percentiles in square brackets for continuous variables and as the percentage for the categorical variables*.

The parameters of SII ≥ 1331.4 × 10^9^, NLR ≥ 3.24, and PLR ≥ 134, produced a corresponding sensitivity of 0.525, 0.711 and 0.231, a specificity of 0.695, 0.584, and 0.216, respectively, for predicting IVIG resistance in KD (Table 2). The AUC value of SII (AUC: 0.607, 95%CI: 0.573–0.640) did not significantly differ from that of NLR (AUC: 0.680, 95%CI: 0.647–0.712) and PLR (AUC: 0.613, 95%CI: 0.579–0.646) (all *p* > 0.05) (Figure 2A).

**Table 2:** Predictive values of SII, NLR, and PLR for IVIG resistance and cardiovascular complications in the whole cohort.

	**AUC**	**SE**	**95%CI**	**Sensitivity**	**Specificity**	**PPV**	**NPV**	**Diagnostic accuracy**	***p-*value**
**IVIG resistance prediction**
^*^SII ≥ 1331.4 × 10^9^	0.607	0.031	0.573–0.640	0.525	0.711	0.231	0.900	0.685	<0.001
NLR ≥ 3.24	0.680	0.027	0.647–0.712	0.695	0.584	0.216	0.920	0.599	<0.001
PLR ≥ 134	0.613	0.031	0.579–0.646	0.500	0.699	0.216	0.894	0.671	<0.001
**Myocarditis prediction**
SII ≥ 1368.6 × 10^9^	0.689	0.0359	0.618–0.759	0.614	0.723	0.169	0.953	0.714	<0.001
^**^NLR ≥ 3.86	0.763	0.0291	0.706–0.820	0.771	0.668	0.175	0.969	0.675	<0.001
**Valve regurgitation prediction**
^#^SII ≥ 1002.4 × 10^9^	0.698	0.0377	0.625–0.772	0.754	0.584	0.126	0.968	0.597	<0.001
NLR ≥ 3.15	0.700	0.0367	0.628–0.772	0.746	0.553	0.105	0.968	0.564	<0.001
**Kawasaki disease shock syndrome prediction**
SII ≥ 1485.4 × 10^9^	0.696	0.0587	0.581–0.811	0.67	0.73	0.08	0.98	0.73	<0.001
^##^NLR ≥ 4.91	0.802	0.0379	0.728–0.877	0.79	0.74	0.10	0.99	0.75	<0.001

*SII, systemic inflammatory index; NLR, neutrophil-lymphocyte ratio; PLR, platelet-lymphocyte ratio; IVIG, intravenous immunoglobulin; NPV, negative predictive value; PPV, positive predictive value*.

* *Pairwise comparison of ROC curves between SII and NLR, PLR in predicting initial IVIG resistance by De Long test, p = 0.396, 0.309*.

** *Pairwise comparison of ROC curves between NLR and SII, PLR in predicting myocarditis by De Long test, p = 0.002 and <0.001*.

# *Pairwise comparison of ROC curves between SII and PLR in predicting valve regurgitation by De Long test, p = 0.037*.

## *Pairwise comparison of ROC curves between NLR and SII, PLR in predicting Kawasaki disease shock syndrome by De Long test, p = 0.022 and 0.004*.

**Figure 2:**
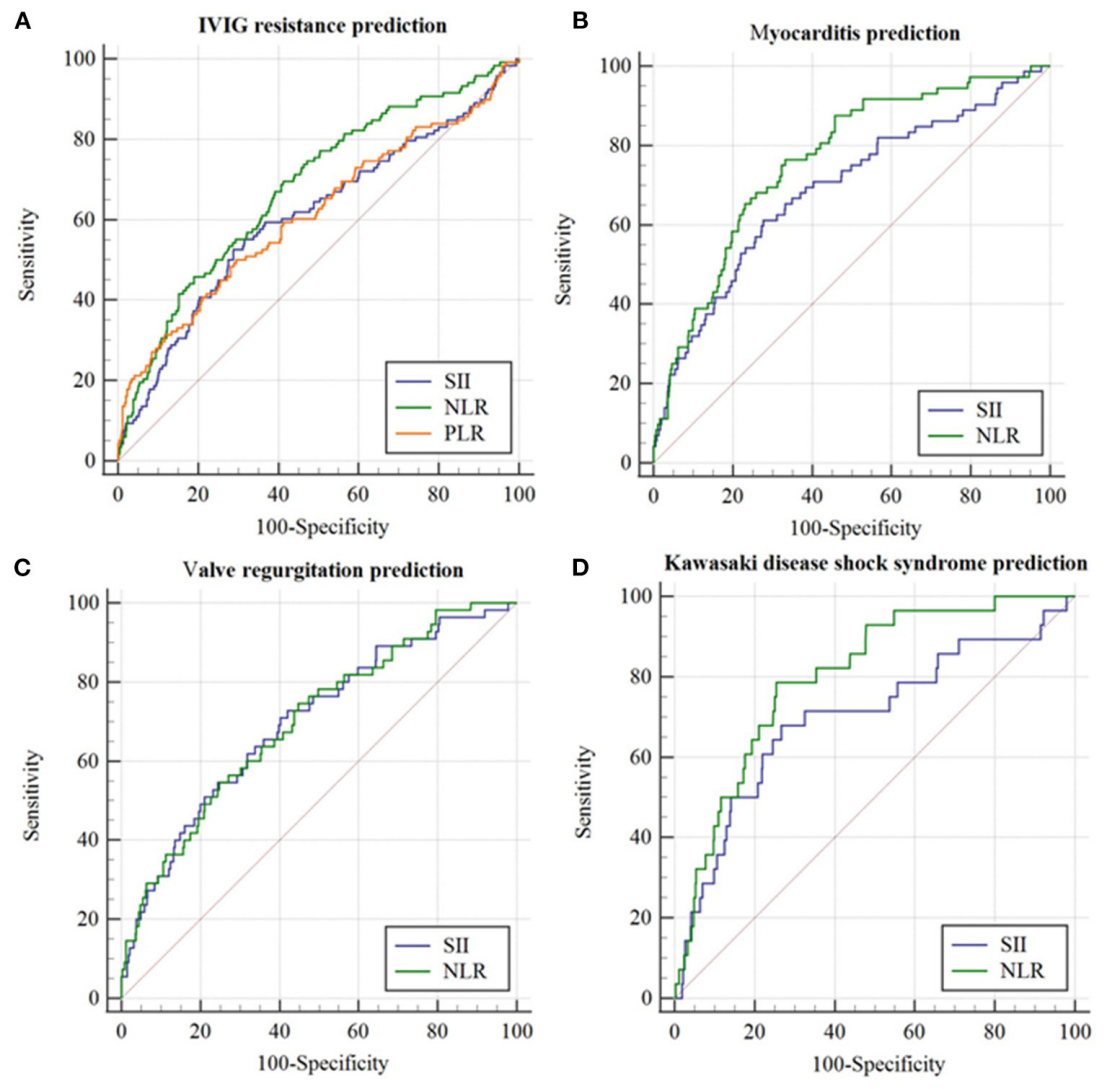
The receiver operating characteristic (ROC) curve for SII, NLR, and PLR in predicting initial IVIG resistance and cardiovascular complications in the whole cohort. **(A)** IVIG resistance prediction; **(B)** Myocarditis prediction; **(C)** Valve regurgitation prediction; **(D)** Kawasaki disease shock syndrome prediction.

### Predictive Value of SII for Myocarditis in Patients With KD

There were 72 patients with myocarditis in the present study. Of them, 61 patients had cardiac enlargement, 17 had arrhythmia (atrial ventricular lock [AVB]: I° AVB, *n* = 15; II° AVB, *n* = 2), 14 had elevated cTnI levels, two had left ventricular dysfunction.

Both SII (1636.8 [812.7–3053.6] × 10^9^ vs. 844.6 [474.9–1506.7] × 10^9^, *p* = 0.001) and NLR (5.87 [3.89–10.91] vs. 2.72 [1.75–4.71], *p* < 0.001) were significantly higher in patients with myocarditis than patients without. After adjusted by age, fever duration before initial IVIG, WBC, CRP, ALT, ALB, TBil, Na^+^, SII was identified as an independent risk factor for myocarditis (*p* = 0.012) (Table 3).

**Table 3:** Comparison of clinical data between the groups of patients with myocarditis and patients without in Kawasaki disease.

	**Patients without myocarditis(*n* = 759)**	**Patients with myocarditis(*n* = 72)**	**Univariate analysis *p*-value**	**Multivariate analysis**	
				**OR (95%CI)**	***p*-value**
Male	420 (55.3)	47 (65.3)	0.108		
Age, years	2.04 (1.08–3.58)	3.33 (2.12–5.00)	<0.001	1.190 (1.043–1.357)	0.010
**Clinical manifestations**					
Rash	596 (78.3)	53 (75.7)	0.544		
Extremity changes	419 (55.2)	43 (59.7)	0.535		
Conjunctivitis	699 (92.1)	62 (86.1)	0.115		
Oral changes	686 (90.4)	67 (93.1)	0.671		
Cervical lymphadenopathy	341 (44.8)	40 (57.1)	0.067		
Fever duration before initial IVIG, days	5.0 (5.0–6.0)	5.0 (4.0–6.0)	0.008	0.781 (0.630–0.967)	0.023
Incomplete KD	291 (38.2)	24 (34.3)	0.607		
IVIG resistance	102 (13.4)	16 (22.9)	0.047		
KDSS	6 (0.8)	22 (30.6)	<0.001		
CALs	71 (9.4)	17 (23.6)	0.001		
**Before initial IVIG**					
The time of blood test, days	5.0 (4.0–6.0)	4.0 (3.0–5.0)	0.041		
WBC, × 10^9^/L	13.2 (10.6–16.6)	14.2 (10.9–17.4)	0.048	1.011 (0.961–1.063)	0681
Neutrophil, %	66.2 (56.0–77.1)	79.8 (72.8–87.4)	<0.001		
Lymphocyte, %	24.3 (16.0–33.0)	13.3 (8.1–19.6)	<0.001		
Hemoglobin, g/L	109.0 (102.0–117.0)	107.5 (102.0–115.0)	0.313		
PLT, × 10^9^/L	314.0 (253.0–383.0)	283.5 (214.5–351.0)	0.034		
CRP, mg/L	72.0 (42.0–107.0)	109.0 (69.8–169.3)	<0.001	1.007 (1.002–1.065)	0.009
ESR, mm/h	65.0 (46.0–81.0)	62.0 (45.5–87.0)	0.827		
AST, U/L	32.0 (25.0–49.0)	35.0 (25.3–73.0)	0.334		
ALT, U/L	36.0 (21.0–82.0)	54.5 (31.3–138.0)	0.042	1.000 (0.997–1.003)	0.862
ALB, g/L	38.0 (35.0–41.0)	36.0 (31.8–38.0)	0.040	0.930 (0.876–0.987)	0.016
TBil, μmol/L	6.0 (4.0–8.7)	6.3 (4.0–15.7)	0.006	1.005 (0.984–1.023)	0.616
Na^+^, mmol/L	137.0 (135.0–139.0)	135.0 (132.8–137.2)	<0.001	0.999 (0.980–1.019)	0.926
NLR	2.72 (1.75–4.71)	5.87 (3.89–10.91)	<0.001		
PLR	99.5 (70.2–148.9)	137.3 (93.8–248.1)	0.123		
SII	844.6 (474.9–1506.7)	1636.8 (812.7–3053.6)	<0.001	1.000 (1.000–1.000)	0.012

*ALB, Albumin; AST, aspartate aminotransferase; ALT, alanine aminotransferase; CRP, C-reactive protein; CALs, coronary artery lesions; ESR, erythrocyte sedimentation rate; IVIG, intravenous immunoglobulin; KD, Kawasaki disease; KDSS, Kawasaki disease shock syndrome; NLR, neutrophil-lymphocyte ratio; PLR, platelet-lymphocyte ratio; TBil, total bilirubin; Na+, sodium; WBC, white blood cell; SII, systemic inflammatory index*.

*The data are presented as the median with the 25 and 75th percentiles in square brackets for continuous variables and as the percentage for the categorical variables*.

The parameter of SII ≥ 1368.6 × 10^9^ and NLR ≥ 3.86 produced a corresponding sensitivity of 0.614 and 0.771, a specificity of 0.723, and 0.668, respectively, for predicting myocarditis in KD (Table 2). The AUC value of SII (AUC: 0.689, 95%CI: 0.618–0.759) was not superior to that of NLR (AUC: 0.763, 95%CI: 0.706–0.820) (*p* = 0.02) (Figure 2B).

### Predictive Value of SII in Valve Regurgitation in Patients With KD

In total, 55 patients with KD were identified as valve regurgitation. Both SII (1723.1 [882.7–3064.0] × 10^9^ vs. 850.8 [474.3–1537.7] × 10^9^, *p* = 0.002) and NLR (5.30 [3.01–10.71] vs. 2.81 [1.75–4.97], *p* = 0.001) were significantly higher in patients with valve regurgitation than patients without. SII was identified as an independent risk factor for valve regurgitation after adjusted by age, the presence of cervical lymphadenopathy, WBC, CRP, ALT, TBil (*p* < 0.001) (Table 4).

**Table 4:** Comparison of clinical data between the groups of patients with and without valve regurgitation in Kawasaki disease.

	**Patients without valve regurgitation(*n* = 776)**	**Patients with valve regurgitation(*n* = 55)**	**Univariate analysis *p*-value**	**Multivariate analysis**	
				**OR (95%CI)**	***p*-value**
Male	435 (56.1)	32 (58.2)	0.781		
Age, years	2.08 (1.13–3.58)	3.29 (2.00–4.81)	<0.001	0.839 (0.719–0.979)	0.026
**Clinical manifestations**					
Rash	607 (78.2)	42 (76.4)	0.747		
Extremity changes	434 (55.9)	28 (50.9)	0.485		
Conjunctivitis	712 (91.8)	49 (6.4)	0.452		
Oral changes	702 (90.5)	51 (92.7)	0.810		
Cervical lymphadenopathy	348 (44.8)	33 (60.0)	0.029	0.931 (0.547–1.585)	0.792
Fever duration before initial IVIG, days	5.0 (5.0–6.0)	5.0 (5.0–6.0)	0.103		
Incomplete KD	293 (37.8)	22 (40.0)	0.774		
IVIG resistance	106 (13.7)	12 (21.8)	0.108		
CALs	76 (9.8)	12 (21.8)	0.011		
**Before initial IVIG**					
The time of blood test	5.0 (4.0–6.0)	4.0 (4.0–6.0)	0.493		
WBC, × 10^9^/L	13.2 (10.6–16.6)	15.3 (12.9–17.6)	<0.001	0.843 (0.808–0.879)	<0.001
Neutrophil, %	67.2 (56.8–77.6)	77.3 (66.1–86.7)	<0.001		
Lymphocyte, %	24.0 (15.1–33.0)	14.3 (8.2–23.3)	<0.001		
Hemoglobin, g/L	109.0 (102.0–117.0)	107.0 (101.0–114.0)	0.211		
PLT, × 10^9^/L	312.0 (252.0–380.8)	306.0 (252.0–376.0)	0.989		
CRP, mg/L	72.0 (43.0–109.0)	103.0 (77.0–133.2)	<0.001	0.996 (0.990–1.001)	0.126
ESR, mm/h	64.0 (46.0–81.0)	67.0 (48.0–88.0)	0.230		
AST, U/L	32.0 (25.0–49.0)	39.0 (27.0–73.0)	0.516		
ALT, U/L	36.0 (21.0–80.5)	67.0 (27.0–130.0)	0.017	0.999 (0.995–1.002)	0.411
ALB, g/L	38.0 (35.0–41.0)	35.2 (32.8–37.3)	0.123		
TBil, μmol/L	6.0 (4.0–8.7)	7.5 (4.0–15.0)	0.020	1.002 (0.979–1.026)	0.855
Na^+^, mmol/L	137.0 (134.8–139.0)	136.4 (134.0–139.0)	0.050		
NLR	2.81 (1.75–4.97)	5.30 (3.01–10.71)	0.001		
PLR	99.8 (70.3–149.9)	128.7 (96.2–224.1)	0.161		
SII	850.8 (474.3–1537.7)	1723.1 (882.7–3064.0)	0.002	1.001 (1.000–1.001)	<0.001

*ALB, Albumin; AST, aspartate aminotransferase; ALT, alanine aminotransferase; CRP, C-reactive protein; CALs, coronary artery lesions; ESR, erythrocyte sedimentation rate; IVIG, intravenous immunoglobulin; KD, Kawasaki disease; KDSS, Kawasaki disease shock syndrome; NLR, neutrophil-lymphocyte ratio; PLR, platelet-lymphocyte ratio; TBil, total bilirubin; Na+, sodium; WBC, white blood cell; SII, systemic inflammatory index*.

*The data are presented as the median with the 25 and 75th percentiles in square brackets for continuous variables and as the percentage for the categorical variables*.

The parameter of SII ≥ 1002.4 × 10^9^ and NLR ≥ 3.15 produced a corresponding sensitivity of 0.754 and 0.746, a specificity of 0.584 and 0.553, respectively, for predicting valve regurgitation in KD (Table 2). The AUC value of SII (AUC:0.698, 95%CI:0.625–0.772, *p* < 0.001) was similar to that of NLR (AUC: 0.700, 95%CI: 0.628–0.772) (*p* > 0.05) (Figure 2C).

### Predictive Value of SII for KDSS in Patients With KD

In the present study, there were 28 patients diagnosed with KDSS. Both SII (1981.1 [780.9–3094.8] × 10^9^ vs. 879.7 [487.5–1576.9] × 10^9^, *p* = 0.001) and NLR (7.47 [4.95–11.91] vs. 2.83 [1.76–5.05], *p* = 0.001) were significantly higher in patients with KDSS than patients without. However, SII was not an independent risk factor for KDSS (*p* = 0.294) after adjusted by age, fever duration before initial IVIG, CRP, ALB, TBil, and Na^+^ (Table 5).

**Table 5:** Comparison of clinical data between the groups of patients with and without Kawasaki disease shock syndrome.

	**Patients without KDSS(*n* = 803)**	**Patients with KDSS(*n* = 28)**	**Univariate analysis *p*-value**	**Multivariate analysis**	
				**OR (95%CI)**	***p-*value**
Male	453 (56.4)	14 (50.0)	0.563		
Age, years	2.08 (1.17–3.58)	3.41 (2.17–4.44)	0.038	1.205 (0.987–1.471)	0.067
**Clinical manifestations**					
Rash	625 (77.8)	24 (85.7)	0.322		
Extremity changes	444 (55.3)	18 (64.3)	0.440		
Conjunctivitis	740 (92.2)	21 (75.0)	0.006		
Oral changes	726 (90.4)	27 (96.4)	0.506		
Cervical lymphadenopathy	363 (45.2)	18 (64.3)	0.046		
Fever duration before initial IVIG, days	5.0 (5.0–6.0)	5.0 (4.0–5.0)	<0.001	0.519 (0.355–0.758)	0.001
IVIG resistance	104 (13.0)	14 (50.0)	<0.001		
Incomplete KD	306 (38.1)	9 (32.1)	0.560		
CALs	79 (9.8)	9 (32.1)	0.001		
**Before initial IVIG**					
Time of blood test	5.0 (4.0–6.0)	3.0 (4.0–5.0)	0.035		
WBC, × 10^9^/L	13.3 (10.6–16.6)	14.9 (11.6–17.2)	0.170		
Neutrophil, %	67.3 (56.9–77.6)	82.1 (75.8–88.6)	<0.001		
Lymphocyte, %	24.0 (15.0–32.3)	11.0 (7.6–15.9)	<0.001		
Hemoglobin, g/L	109.0 (102.0–116.0)	107.5 (102.0–116.8)	0.663		
PLT, × 10^9^/L	312 (253–382)	273 (138–345)	0.029		
CRP, mg/L	73 (43.3–111.0)	102.5 (68.5–129.8)	0.039	1.002 (0.993–1.011)	0.642
ESR, mm/h	65.0 (46.0–81.0)	57.5 (43.0–78.8)	0.481		
AST, U/L	32.0 (25.0–49.0)	58.0 (28.8–95.8)	0.112		
ALT, U/L	37.0 (21.0–83.0)	85.5 (27.0–151.0)	0.064		
ALB, g/L	38.0 (35.0–41.0)	36.0 (30.4–39.6)	0.016	0.944 (0.863–1.033)	0.212
TBil, μmol/L	6.0 (4.0–8.7)	12.0 (4.0–32.0)	0.007	1.025 (1.005–1.046	0.015
Na^+^, mmol/L	137.0 (134.9–139.0)	134.5 (133.0–136.6)	<0.001	1.006 (0.978–1.034)	0.679
NLR	2.83 (1.76–5.05)	7.47 (4.95–11.91)	0.001		
PLR	101.5 (70.9–150.0)	159.3 (86.9–264.1)	0.376		
SII	879.7 (487.5–1576.9)	1981.1 (780.9–3094.8)	0.001	1.000 (1.000–1.000)	0.294

*ALB, Albumin; AST, aspartate aminotransferase; ALT, alanine aminotransferase; CRP, C-reactive protein; CALs, coronary artery lesions; ESR, erythrocyte sedimentation rate; IVIG, intravenous immunoglobulin; KD, Kawasaki disease; KDSS, Kawasaki disease shock syndrome; NLR, neutrophil-lymphocyte ratio; PLR, platelet-lymphocyte ratio; TBil, total bilirubin; Na+, sodium; WBC, white blood cell; SII, systemic inflammatory index*.

*The data are presented as the median with the 25 and 75th percentiles in square brackets for continuous variables and as the percentage for the categorical variables*.

The parameter of SII ≥ 1485.4 × 10^9^ and NLR ≥ 4.91 produced a corresponding sensitivity of 0.67 and 0.79, a specificity of 0.73 and 0.74, respectively, for KDSS prediction in KD (Table 2). The AUC value of SII (AUC:0.696, 95%CI:0.581–0.811, *p* < 0.001) was not superior to that of NLR (AUC: 0.700, 95%CI: 0.628–0.772) (all *p* = 0.022) (Figure 2D).

### Predictive Value of SII for CALs and Pericardial Effusion in Patients With KD

There was no significant difference of SII between patients with CALs and patients with non-CALs (857.7 [508.7–1598.1] × 10^9^ vs. 884.1 [495.3–1606.9] × 10^9^, *p* = 0.559), as well as patients with pericardial effusion and those without (880.3 [486.6–1578.7] × 10^9^ vs. 1723.1 [723.9–2470.4] × 10^9^, *p* = 0.056). Additionally, during the follow-up (at 1 month, 6 months, and 12 months after disease onset), SII in patients with CALs also did not significantly differ from those without CALs (one month: 1012.2 [549.5–1767.1] × 10^9^ vs. 882.6 [495.3–1599.8] × 10^9^, *p* = 0.176; 6 months: 971.8 [185.3–1836.0] × 10^9^ vs. 883.1 [495.5–1601.6] × 10^9^, *p* = 0.830; 12 months: 971.8 [627.3–1836.0] × 10^9^ vs. 883.1 [495.3–1603.9] × 10^9^, *p* = 0.696).

### Predictive Abilities of SII for IVIG Resistance and Cardiovascular Complications in Patients With KD Stratified by PLT Level

The study participants were divided into the thrombocytopenia group (PLT <150 × 10^9^/L, *n* = 81) and the non-thrombocytopenia group (PLT ≥ 150 × 10^9^/L, *n* = 750). Further analysis of predictive values of SII for IVIG resistance and cardiovascular complications in KD patients stratified by PLT level was performed, and the results were illustrated in Supplementary Material 1. In the thrombocytopenia group, there were no significant differences in the values of SII, NLR, and PLR between IVIG responders and non-responders, as well as between patients with cardiovascular complications and those without (all *p* > 0.05).

**Figure 3:**
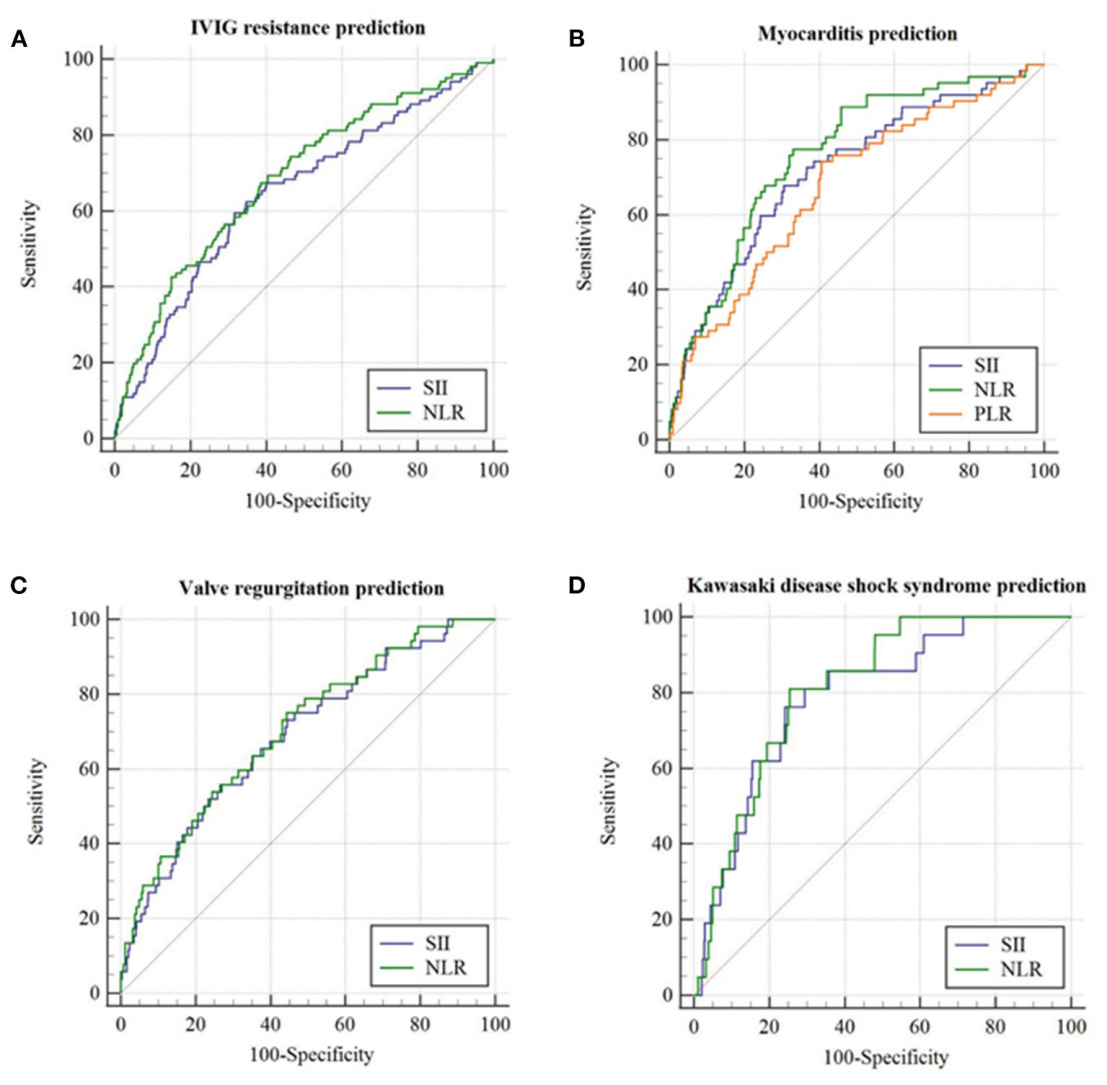
The receiver operating characteristic (ROC) curve for SII, NLR, and PLR in predicting initial IVIG resistance and cardiovascular complications in patients with KD having PLT ≥ 150 × 10^9^/L. **(A)** IVIG resistance prediction; **(B)** Myocarditis prediction; **(C)** Valve regurgitation prediction; **(D)** Kawasaki disease shock syndrome prediction.

In the non-thrombocytopenia group, a significant difference was evidenced in SII between IVIG responders and non-responders (942.0 [576.9–1597.2] × 10^9^ vs. 1499.1 [771.4–2419.9] × 10^9^, *p* = 0.001). The best cutoff value of SII for predicting IVIG resistance in the non-thrombocytopenia group was 1331.4 × 10^9^, yielding a sensitivity, specificity, PPV, NPV of 0.59, 0.68, 0.226, 0.915, respectively. The predictive ability of which was not much better than that of SII ≥ 1331.4 × 10^9^ in the total group (Table 6 and Figure 3A).

**Table 6:** Predictive ability of SII, NLR, and PLR in patients with KD with PLT levels of ≥150 × 10^9^/L.

	**AUC**	**SE**	**95%CI**	**Sensitivity**	**Specificity**	**PPV**	**NPV**	**Diagnostic accuracy**	***p-*value**
**IVIG resistance prediction**
SII ≥ 1331.4 × 10^9^	0.645	0.0307	0.585–0.706	0.59	0.68	0.226	0.915	0.671	<0.001
NLR ≥ 3.27	0.684	0.0293	0.627–0.742	0.69	0.60	0.210	0.926	0.608	<0.001
**Myocarditis prediction**
SII ≥ 1368.6 × 10^9^	0.718	0.0352	0.650–0.787	0.68	0.69	0.167	0.959	0.693	<0.001
NLR ≥ 3.86	0.761	0.0306	0.701–0.821	0.77	0.67	0.174	0.972	0.677	<0.001
PLR≥119.5	0.676	0.0362	0.605–0.747	0.74	0.59	0.142	0.962	0.607	<0.001
**Valve regurgitation prediction**
SII ≥ 1574.3 × 10^9^	0.690	0.0383	0.615–0.765	0.56	0.73	0.133	0.957	0.717	<0.001
NLR ≥ 3.15	0.706	0.0372	0.633–0.779	0.75	0.56	0.112	0.968	0.569	<0.001
**KDSS prediction**
SII ≥ 1700.6 × 10^9^	0.792	0.0453	0.703–0.880	0.76	0.76	0.083	0.991	0.757	<0.001
NLR ≥ 4.91	0.813	0.0361	0.742–0.884	0.81	0.75	0.084	0.993	0.747	<0.001

*SII, systemic inflammatory index; NLR, neutrophil-lymphocyte ratio; PLR, platelet-lymphocyte ratio; PLT, platelet; IVIG, intravenous immunoglobulin; KDSS, Kawasaki disease shock syndrome; NPV, negative predictive value; PPV, positive predictive value*.

In terms of cardiovascular complications in the non-thrombocytopenia group, there was a significant difference in the values of SII between the myocarditis group and non-myocarditis group (1784.5 [1061.1–3184.7] × 10^9^ vs. 951.8 [578.8–1598.5] × 10^9^, *p* = 0.001). The best cutoff value of SII for predicting myocarditis in the non-thrombocytopenia group was 1368.6 × 10^9^ with an AUC of 0.718 (95%CI: 0.650–0.787), yielding a sensitivity, specificity, PPV, NPV of 0.68, 0.69, 0.167, 0.959, 0.693, respectively, the predictive ability of which were similar to that of SII ≥ 1368.6 × 10^9^ in the total group (Table 6 and Figure 3B).

In comparison with non-valve regurgitation group, patients with valve regurgitation had significant higher SII (1743.2 [917.9–3053.6] × 10^9^ vs. 961.2 [582.5–1615.2] × 10^9^, *p* = 0.004) with an AUC of 0.690 (95%CI: 0.615–0.765), producing a sensitivity, specificity, PPV, NPV of 0.56, 0.73, 0.133, 0.957, 0.717, respectively (Table 6 and Figure 3C). The predictive value of SII for valve regurgitation in the non-thrombocytopenia group was not better than that of SII ≥ 1002.4 × 10^9^ in the total group.

The SII between KDSS group and non-KDSS group was also significantly different (2308.5 [1597.9–3579.1] × 10^9^ vs. 968.2 [589.0–1643.5] × 10^9^, *p* < 0.001). For KDSS prediction, SII ≥ 1700.6 × 10^9^ had an AUC of 0.792 (95%CI: 0.703–0.880), with a corresponding sensitivity, specificity, PPV, NPV of 0.76, 0.76, 0.083, 0.991, 0.757, respectively (Table 6 and Figure 3D). It seemed to have a better sensitivity than that of SII ≥ 1485.4 × 10^9^ in the total group.

## Discussion

Clinically, IVIG resistance and cardiovascular complications prediction are critical issues in KD. In the present study, with a large dataset, we firstly and prospectively explored the prognostic role of SII for IVIG resistance and cardiovascular complications prediction in KD and compared its predictive value with that of NLR and PLR. Several novel findings were observed: (1) SII was an independent risk factor of IVIG resistance and several cardiovascular complications, and it might provide some references for clinical management, particularly for the prediction of myocarditis and valve regurgitation in KD; (2) Incorporation of platelets into NLR (SII) did not yield better predictive validity in the context of KD; (3) SII might not be applicable for prediction of IVIG resistance and cardiovascular sequelae in KD patients complicated by thrombocytopenia; (4) NLR was effective in predicting cardiovascular complications including myocarditis, valve regurgitation and KDSS in KD, with both high sensitivities and specificities.

Inconsistent with our hypothesis, we observed that the incorporation of platelets into NLR (SII) did not yield better predictive validity in the context of KD. Several explanations were hypothesized. Opposite with chronic inflammation as a mechanism of immune response in cancers (33) where the platelet and neutrophil increased while lymphocytes decreased concurrently (34), the time of peak level of neutrophils, platelet, and lymphocytes were non-synchronous during the acute phase of patients with KD. It has been proved that neutrophil and lymphocyte, separately involve in the first (at 2–10 days from disease onset) and second (at 11–21 days from disease onset) pathological process of KD as acute systemic inflammations (11). Meanwhile, the platelet count begins to elevate approximately at 6 days of the natural course of KD (35), and its peak time occurred 2–3 weeks after disease onset (36). Furthermore, prior studies demonstrated platelet levels during the acute phase tend to decrease in patients with severe KD; a platelet count of <300 × 10^9^/L is included in some risk-scoring systems of IVIG resistance in KD (6, 7). In the present study, it was also found the platelet counts were significantly lower in KD patients with IVIG resistance and/or cardiovascular complications. The time of blood test in our study was 5 (IQR: four-six) days from disease onset when the platelet count remained unchanged or slightly elevated. We speculated that our findings that the values of SII was not superior to NLR in the prediction of IVIG resistance and several cardiovascular complications in KD, could possibly and partly explained by the different kinetics and dynamic change of neutrophils, lymphocytes, and platelet in a different stage of KD.

CALs is not the only cardiovascular abnormalities in patients with KD. Other cardiovascular sequelae like myocarditis, valvular abnormalities, pericarditis, and KDSS are now being increasingly recognized (37). Early identification and appropriate treatment of these complications is also of paramount importance and could improve the prognosis of KD patients. In the present study, we firstly explored the predictive role of SII, NLR, and PLR in cardiovascular complications. It was found both SII and NLR were significantly higher in patients with IVIG resistance, as well as myocarditis, valve regurgitation, and KDSS. Notably, we firstly documented that both SII and NLR could be powerful indicators for the occurrence of myocarditis and KDSS with high sensitivities and specificities. Nonetheless, no significant difference in SII/NLR was evidenced between patients with CALs and patients with non-CALs. Previous findings suggested that a persistent and ongoing inflammatory reaction might be more likely associated with the development of CALs. In comparison with our baseline SII/NLR, their fluctuations might possess greater predictive power for CALs in patients with KD. Therefore, further study might collect different time points of SII/NLR to elevate its predictive ability and prognosis of CALs.

As a novel systemic inflammatory index merging the information of lymphocyte, neutrophil, and platelet counts, the parameter of SII would be theoretically a powerful prognostic indicator or predictor under conditions such as systematic infection and inflammation where the elevated count of neutrophil and platelet, as well as decreased level of lymphocytes, were observed simultaneously. Several studies have proved that SII was more valuable and accurate than either NLR or PLR in prognosis prediction for various types of cancers (19, 22, 23). However, it should be pointed out that, on the basis of the above theory, SII might not be applicable in patients complicated by thrombocytopenia since SII in these patients would be lower. As a severe complication, macrophage activation syndrome (MAS) could occur in the context of KD and always suffered from a higher risk of IVIG resistance and cardiovascular complications (38–43). Thrombocytopenia was a prominent manifestation of MAS in KD (40, 44). A recent study conducted by Rakesh Kumar Pilania et al. from India recorded thrombocytopenia in 11/12 (91.7%) KD patients complicated by MAS during the course of the illness (44). Our study proved that for KD patients with thrombocytopenia (PLT <150 × 10^9^/L), there were no significant differences in the values of SII between KD patients with IVIG resistance/cardiovascular complications and those without, suggesting the limited value of SII in predicting the occurrence of MAS in KD (Supplementary Material 1).

KD is a self-limiting systematic vasculitis that predominantly affects children aged between 6 months and 4 years (1). However, no age group seems to be exempt from developing KD. Many studies aimed to investigate the clinical features of KD outside the usual age range. Despite the criteria for the definition of older children varied greatly among different studies, the conclusion was relatively consistent that older children had an increased risk of IVIG resistance and coronary artery sequalae (45–49). For instance, a recent study conducted by Ankur Kumar Jindal from Northwest India found that IVIG resistance, CALs, myocarditis, and Kawasaki disease shock syndrome (KDSS) were common in children older than 10 years old (45). Our findings agreed with these studies that older age was a risk factor for the occurrence of IVIG resistance and cardiovascular complications, including myocarditis, valve regurgitation as well as KDSS (45–48, 50). Delay in diagnosis and initiation of standard treatment has been considered to be the most important risk factor for the development of IVIG resistance and cardiovascular complications in this age group (45, 47, 48, 51). However, a more marked inflammatory response in older children has also been reported (47). A positive correlation between SII/NLR and age was observed in the present study (Supplementary Material 2). The value of SII and NLR increased significantly after 4 years old. These findings provided additional evidences that a more severe inflammatory reaction reflected by higher SII/NLR in older children may in part contribute to the higher risk of IVIG resistance and cardiovascular complications in this age group.

The limitations of this study included that selection bias may occur as this study was performed in a single institution. The findings might be only applicable to KD patients receiving the standardized IVIG treatment (2 g/Kg) prior to 10 days from fever onset. In addition, SII might not be applicable in patients with thrombocytopenia in KD. Furthermore, t the dynamic variability of SII during the disease course of KD should be taken into consideration because we found the value of SII did not differ between the IVIG responders and non-responders when the blood collection time was 6 days later from fever onset (Supplementary Material 3). Despite the aforementioned limitations, the present study is firstly to determine the predictive value of SII for IVIG resistance and cardiovascular complications with large sample size and prospective approach. It was identified that SII was significantly higher in patients with IVIG resistance and some cardiovascular complications but may only serve as a complementary laboratory marker in the context of KD. It was nothing less but definitely nothing more. Due to an unknown origin of KD and in light of the above findings, we speculate a prediction model combined with other specific indicators rather than clinical and routine laboratory variables might have a better outcome.

## Conclusions

A higher SII was an independent risk factor for initial IVIG resistance, myocarditis and valve regurgitation. Although it may predict IVIG resistance and some cardiovascular complications in KD as a single parameter, its predictive ability was not good enough and not superior to NLR. The dynamic variability of SII during the disease course of KD should be taken into consideration when aiming at predicting IVIG resistance cardiovascular complications. SII might not be applicable in patients with KD having thrombocytopenia. NLR might be a promising indicator for the prediction of myocarditis and KDSS in KD.

## Data Availability Statement

The original contributions generated for this study are included in the article/Supplementary Material, further inquiries can be directed to the corresponding author/s.

## Ethics Statement

The studies involving human participants were reviewed and approved by The University Ethics Committee on Human Subjects at Sichuan University. Written informed consent to participate in this study was provided by the participants' legal guardian/next of kin.

## Author Contributions

XL obtained financial support, collected and analyzed data, and drafted the manuscript. SS collected data, provided Tables 1, 3. LW collected data and provided figures. NZ collected data and analyzed data. MW, LL, and LZ contributed to data collection. YH treated patients at admission, designed the study, and obtained financial support. KZ designed the study and provided Tables 4, 5. CL obtained financial support, analyzed data, and provided Tables 2, 6. YD analyzed data and provided Supplementary Materials. CW conceived and designed the study, obtained financial support, and collected data. The final version of the manuscript was approved by all authors.

## Conflict of Interest

The authors declare that the research was conducted in the absence of any commercial or financial relationships that could be construed as a potential conflict of interest.

## Publisher's Note

All claims expressed in this article are solely those of the authors and do not necessarily represent those of their affiliated organizations, or those of the publisher, the editors and the reviewers. Any product that may be evaluated in this article, or claim that may be made by its manufacturer, is not guaranteed or endorsed by the publisher.

## Abbreviations

AHA: American Heart Association
ALB: Albumin
ALT: Alanine aminotransferase
AST: Aspartate aminotransferase
AUC: Area under the curve
BUN: Blood urea nitrogen
BSA: body surface area
CBC: Complete blood count
CRP: C-reactive protein
CALs: Coronary artery lesions
ESR: Erythrocyte sedimentation rate
IVIG: Intravenous immunoglobulin
KD: Kawasaki disease
KDSS: Kawasaki disease shock syndrome
NPV: Negative predictive value
NLR: Neutrophil-to-lymphocyte ratio
SII: Systemic immune-inflammation index
Na^+^: Sodium
ORs: Odds ratios
PLR: Platelet-to-lymphocyte ratio
PPV: Positive predictive value
ROC: Receiver operating characteristic
SD: Standard deviation
TBil: Total bilirubin
WBC: White blood cell.
