# Supplementary material for: Predictive Value of the Systemic Immune-Inflammation Index for Intravenous Immunoglobulin Resistance and Cardiovascular Complications in Kawasaki Disease
Source: Front Cardiovasc Med. 2021 Aug 24;8:711007. doi: 10.3389/fcvm.2021.711007 (PMC8421732; doi:10.3389/fcvm.2021.711007)
Supplement: Supplementary file 1 [file Data_Sheet_1.DOCX]

**Supplementary material 1**

**Comparison of PLT, NLR, SII, and PLR between** **IVIG resistant and IVIG responsive groups with different platelet levels.**

|  |  | **PLT<150****×10^9^/L，n=81** | ***p* value** | **PLT ≥150×10^9^/L，n=750** | ***p* value** |
| --- | --- | --- | --- | --- | --- |
| **SII**, ×10^9^ |  | 113.2(63.1-237.6) |  | 981.8(593.9-1730.4) |  |
|  | IVIG resistance | N=17  167.6(99.7-592.5) | 0.165 | N=101  1499.1(771.4-2419.9) | 0.001 |
|  | IVIG response | N=64  98.2(56.6-186.8) |  | N=649  942.0(576.9-1597.2) |  |
| **NLR** |  | 2.96(1.78-5.71) |  | 2.95(1.78-5.25) |  |
|  | IVIG resistance | N=17  3.77(2.64-9.07) | 0.174 | N=101  4.81(2.73-8.48) | <0.001 |
|  | IVIG response | N=64  2.79(1.57-5.09) |  | N=649  2.69(1.74-4.69) |  |
| **PLR** |  | 12.9(8.2-24.0) |  | 109.4(80.7-159.2) |  |
|  | IVIG resistance | N=17  18.4(12.1-59.7) | 0.111 | N=101  146.9(95.2-243.2) | 0.068 |
|  | IVIG response | N=64  12.1(7.2-20.5) |  | N=649  105.8(79.0-151.9) |  |

Abbreviations: SII, systemic inflammatory index; NLR, neutrophil-lymphocyte ratio; PLR, platelet-lymphocyte ratio; PLT, platelet; IVIG, intravenous immunoglobulin.

**Comparison of PLT, NLR, SII, and PLR between** **myocarditis and non-myocarditis group with different platelet levels.**

|  |  | **PLT<150×10^9^/L, n=81** | ***p* value** | **PLT ≥150×10^9^/L, n=750** | ***p* value** |
| --- | --- | --- | --- | --- | --- |
| **SII**, ×10^9^ |  | 113.2(63.1-237.6) |  | 981.8(593.9-1730.4) |  |
|  | Myocarditis | N=10  431.1(156.6-1224.3) | 0.089 | N=62  1784.5(1061.1-3184.7) | 0.001 |
|  | Non- Myocarditis | N=71  102.8(62.8-170.9) |  | N=688  951.8(578.8-1598.5) |  |
| **NLR** |  | 2.96(1.78-5.71) |  | 2.95(1.78-5.25) |  |
|  | Myocarditis | N=10  8.37(3.34-12.39) | 0.078 | N=62  5.83(3.93-10.59) | <0.001 |
|  | Non- Myocarditis | N=71  2.84(1.58-4.89) |  | N=688  2.72(1.75-4.70) |  |
| **PLR** |  | 12.9(8.2-24.0) |  | 109.4(80.7-159.2) |  |
|  | Myocarditis | N=10  67.8(20.9-133.6) | 0.031 | N=62  150.4(112.5-261.6) | 0.136 |
|  | Non- Myocarditis | N=71  12.2(7.9-18.4) |  | N=688  106.3(79.8-154.4) |  |

Abbreviations: SII, systemic inflammatory index; NLR, neutrophil-lymphocyte ratio; PLR, platelet-lymphocyte ratio; PLT, platelet; IVIG, intravenous immunoglobulin.

**Comparison of PLT, NLR, SII, and PLR between** **valve regurgitation and non- valve regurgitation group with different platelet levels.**

|  |  | **PLT<150×10^9^/L, n=81** | ***p* value** | **PLT ≥150×10^9^/L, n=750** | ***p* value** |
| --- | --- | --- | --- | --- | --- |
| **SII**, ×10^9^ |  | 113.2(63.1-237.6) |  | 981.8(593.9-1730.4) |  |
|  | valve regurgitation | N=3  167.6(53.31-4025.90) | 0.455 | N=52  1743.2(917.9-3053.6) | 0.004 |
|  | Non- valve regurgitation | N=78  111.7(63.2-227.7) |  | N=698  961.2(582.5-1615.2) |  |
| **NLR** |  | 2.96(1.78-5.71) |  | 2.95(1.78-5.25) |  |
|  | valve regurgitation | N=3  5.641.55-31.70) | 0.468 | N=52  5.19(3.05-10.65) | 0.001 |
|  | Non- valve regurgitation | N=78  2.94(1.79-5.39) |  | N=698  2.78(1.75-4.92) |  |
| **PLR** |  | 12.9(8.2-24.0) |  | 109.4(80.7-159.2) |  |
|  | valve regurgitation | N=3  13.3(N/A) | 0.483 | N=52  131.8(100.2-222.5) | 0.006 |
|  | Non- valve regurgitation | N=78  12.9(8.2-23.7) |  | N=698  106.8(79.9-154.7) |  |

Abbreviations: SII, systemic inflammatory index; NLR, neutrophil-lymphocyte ratio; PLR, platelet-lymphocyte ratio; PLT, platelet; IVIG, intravenous immunoglobulin.

**Comparison of PLT, NLR, SII, and PLR between KDSS and non-KDSS group with different platelet levels.**

|  |  | **PLT<150×10^9^/L, n=81** | ***p* value** | **PLT ≥150×10^9^/L，n=750** | ***p* value** |
| --- | --- | --- | --- | --- | --- |
| **SII**, ×10^9^ |  | 113.2(63.1-237.6) |  | 981.8(593.9-1730.4) |  |
|  | KDSS | N=7  557.7(167.6-1586.0) | 0.168 | N=21  2308.5(1597.9-3579.1) | <0.001 |
|  | Non-KDSS | N=74  106.9(62.9-180.6) |  | N=729  968.2(589.0-1643.5) |  |
| **NLR** |  | 2.96(1.78-5.71) |  | 2.95(1.78-5.25) |  |
|  | KDSS | N=7  8.58(3.25-15.30) | 0.128 | N=21  6.96(4.98-11.18) | <0.001 |
|  | Non-KDSS | N=74  2.86(1.72-5.16) |  | N=729  2.82(1.76-5.03) |  |
| **PLR** |  | 12.9(8.2-24.0) |  | 109.4(80.7-159.2) |  |
|  | KDSS | N=7  52.9(13.3-154.8) | 0.128 | N=21  182.2(127.2-284.4) | 0.294 |
|  | Non-KDSS | N=74  12.6(8.1-21.4) |  | N=729  107.9(80.0-155.2) |  |

Abbreviations: SII, systemic inflammatory index; NLR, neutrophil-lymphocyte ratio; PLR, platelet-lymphocyte ratio; PLT, platelet; IVIG, intravenous immunoglobulin; NPV, negative predictive value; PPV, positive predictive value.
