# Supplementary material for: Predictive Value of the Systemic Immune-Inflammation Index for Intravenous Immunoglobulin Resistance and Cardiovascular Complications in Kawasaki Disease
Source: Front Cardiovasc Med. 2021 Aug 24;8:711007. doi: 10.3389/fcvm.2021.711007 (PMC8421732; doi:10.3389/fcvm.2021.711007)
Supplement: Supplementary file 2 [file Data_Sheet_2.DOCX]

**Supplementary materials 2**

**The dynamic variability of PLT, NLR, SII, and PLR in the patients with age.**

| **Age (years)** | **Patients number** | **SII, ×10^9^** | **PLT, ×10^9^** | **NLR** | **PLR** |
| --- | --- | --- | --- | --- | --- |
| 0~0.5 | 58 | 676.9(397.0-1219.3) | 354 (265-471) | 1.95(1.29-3.91) | 86.4(63.2-132.9) |
| 0.5~1.0 | 98 | 640.9(377.2-1006.2) | 355(264-454) | 1.86(1.32-2.68) | 71.9(54.2-105.2) |
| 1.0~2.0 | 222 | 663.4(381.9-1001.0) | 309(254-370) | 2.19(1.51-3.15) | 88.9(66.5-116.6) |
| 2.0~3.0 | 164 | 1057.8(612.0-1586.4) | 317(261-379) | 3.25(2.14-5.18) | 109.8(81.2-154.3) |
| 3.0~4.0 | 121 | 1149.9(605.1-2198.8) | 300(245-362) | 4.23(2.63-7.15) | 120.8(91.3-181.8) |
| 4.0~5.0 | 78 | 1351.7(795.4-2235.9) | 297(253-365) | 4.85(2.64-7.81) | 144.7(98.9-201.6) |
| 5.0~7.0 | 63 | 1942.5(644.4-3144.6) | 294(225-339) | 6.98(4.21-10.57) | 169.9(92.1-260.9) |
| 7.0~ | 27 | 2118.7(1196.3-3088.8) | 279(196-353) | 7.56(5.37-12.99) | 159.9(121.1-269.6) |

Abbreviations: SII, systemic inflammatory index; NLR, neutrophil-lymphocyte ratio; PLR, platelet-lymphocyte ratio; PLT, platelet; IVIG, intravenous immunoglobulin.

^*^ *p*<0.05; ^ns^ *p*>0.05;

**
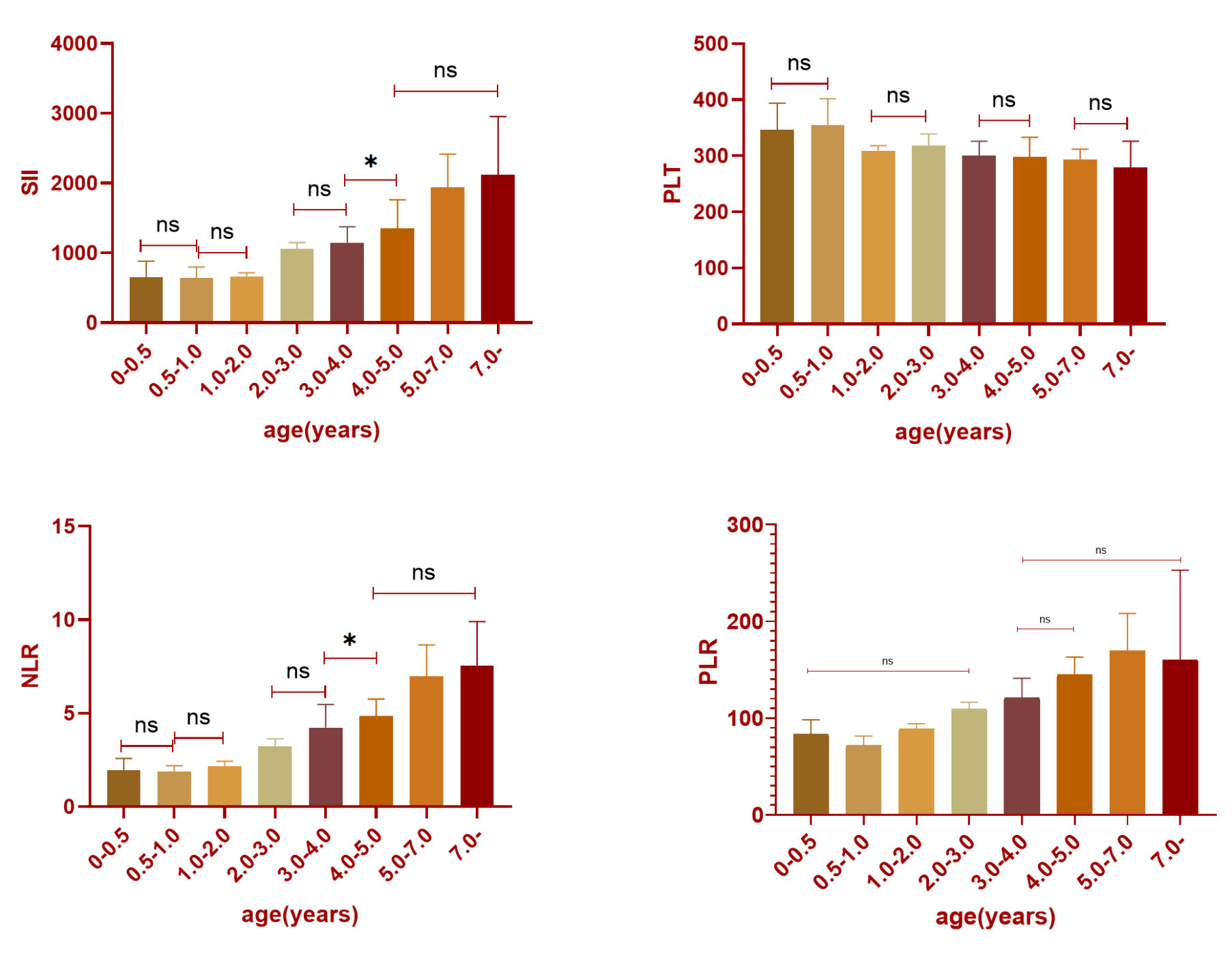
**
