# Supplementary material for: Predictive Value of the Systemic Immune-Inflammation Index for Intravenous Immunoglobulin Resistance and Cardiovascular Complications in Kawasaki Disease
Source: Front Cardiovasc Med. 2021 Aug 24;8:711007. doi: 10.3389/fcvm.2021.711007 (PMC8421732; doi:10.3389/fcvm.2021.711007)
Supplement: Supplementary file 3 [file Data_Sheet_3.DOCX]

**Supplementary material 3**

**Comparison of PLT, NLR, SII, and PLR between IVIG resistant and IVIG responsive groups at different blood sampling days.**

|  |  | **2-4 days** | ***p-*value** | **5 days** | ***p* value** | **6 days** | ***p-*value** | **≥7 days** | ***p-*value** |
| --- | --- | --- | --- | --- | --- | --- | --- | --- | --- |
| **PLT**  ×10^9^ |  | 302(249-362) |  | 312(255-373) |  | 317(249-382) |  | 373(290-459) |  |
|  | IVIG resistance | N=63  296(228-346) | 0.105 | N=26  274(187-321) | 0.006 | N=10  162(32-280) | 0.004 | N=19  306(281-442) | 0.192 |
|  | IVIG response | N=349  306(251-365) |  | N=174  316(260-385) |  | N=99  325(264-388) |  | N=91  381(297-466) |  |
| **NLR** |  | 3.15(1.97-5.61) |  | 3.11(1.77-5.63) |  | 3.02(1.75-5.17) |  | 2.01(1.40-3.55) |  |
|  | IVIG resistance | N=64  4.52(2.49-9.26) | ＜0.001 | N=26  6.69(4.30-8.69) | ＜0.001 | N=10  6.57(3.67-14.59) | 0.002 | N=19  2.70(1.33-3.52) | 0.535 |
|  | IVIG response | N=349  2.96(1.88-5.09) |  | N=174  2.62(1.69-4.71) |  | N=99  2.83(1.63-4.56) |  | N=91  1.96(1.41-3.65) |  |
| **SII**  ×10^9^ |  | 961.6(522.6-1695.0) |  | 849.0(526.6-1610.4) |  | 854.6(470.5-1607.5) |  | 775.0(398.3-1350.0) |  |
|  | IVIG resistance | N=64  1370.2(590.0-2444.4) | 0.020 | N=26  1410.8(777.9-2201.3) | 0.010 | N=10  1485.0(159.7-3223.9) | 0.413 | N=19  731.6(379.1-1587.5) | 0.877 |
|  | IVIG response | N=349  883.3(489.6-1577.6) |  | N=174  795.1(499.6-1533.2) |  | N=99  850.9(474.1-1379.3) |  | N=91  782.6(399.8-1310.0) |  |
| **PLR** |  | 105.0(75.1-158.8) |  | 98.2(68.6-150.2) |  | 104.1(66.3-144.2) |  | 99.4(67.9-151.0) |  |
|  | IVIG resistance | N=64  153.8(89.8-302.9) | ＜0.001 | N=26  112.7(78.5-148.1) | 0.128 | N=10  119.3(17.2-304.8) | 0.482 | N=19  94.9(55.7-147.5) | 0.566 |
|  | IVIG response | N=349  99.9(72.9-148.8) |  | N=174  95.6(67.1-148.4) |  | N=99  104.1(67.1-133.6) |  | N=91  103.9(68.6-151.7) |  |

Abbreviations: SII, systemic inflammatory index; NLR, neutrophil-lymphocyte ratio; PLR, platelet-lymphocyte ratio; PLT, platelet; IVIG, intravenous immunoglobulin.

**The dynamic variability of SII, PLT, NLR, and PLR in IVIG resistance prediction**

|  | **Cut-off value** | **AUC** | **SE** | **95%CI** | **Sensitivity** | **Specificity** | ***p-*value** |
| --- | --- | --- | --- | --- | --- | --- | --- |
| **SII**, ×10^9^ |  |  |  |  |  |  |  |
| 2-4 days | ≥ 1331.4 | 0.593 | 0.0435 | 0.544-0.641 | 0.546 | 0.693 | 0.032 |
| 5 days | ≥1113.8 | 0.658 | 0.0574 | 0.587-0.723 | 0.692 | 0.649 | 0.006 |
| **PLT**, ×10^9^ |  |  |  |  |  |  |  |
| 5 days | ≤294 | 0.667 | 0.0515 | 0.597-0.732 | 0.654 | 0.644 | 0.001 |
| 6 days | ≤196 | 0.775 | 0.0927 | 0.685-0.849 | 0.700 | 0.859 | 0.003 |
| **NLR** |  |  |  |  |  |  |  |
| 2-4 days | >3.27 | 0.668 | 0.0382 | 0.620 -0.713 | 0.682 | 0.567 | 0.001 |
| 5 days | >3.94 | 0.782 | 0.0423 | 0.719-0.837 | 0.808 | 0.691 | <0.001 |
| 6 days | >3.37 | 0.797 | 0.0686 | 0.709 -0.868 | 0.900 | 0.616 | <0.001 |
| **PLR** |  |  |  |  |  |  |  |
| 2-4 days | >180.4 | 0.676 | 0.0418 | 0.628-0.721 | 0.444 | 0.857 | <0.001 |

Abbreviations: SII, systemic inflammatory index; NLR, neutrophil-lymphocyte ratio; PLR, platelet-lymphocyte ratio; PLT, platelet; IVIG, intravenous immunoglobulin.


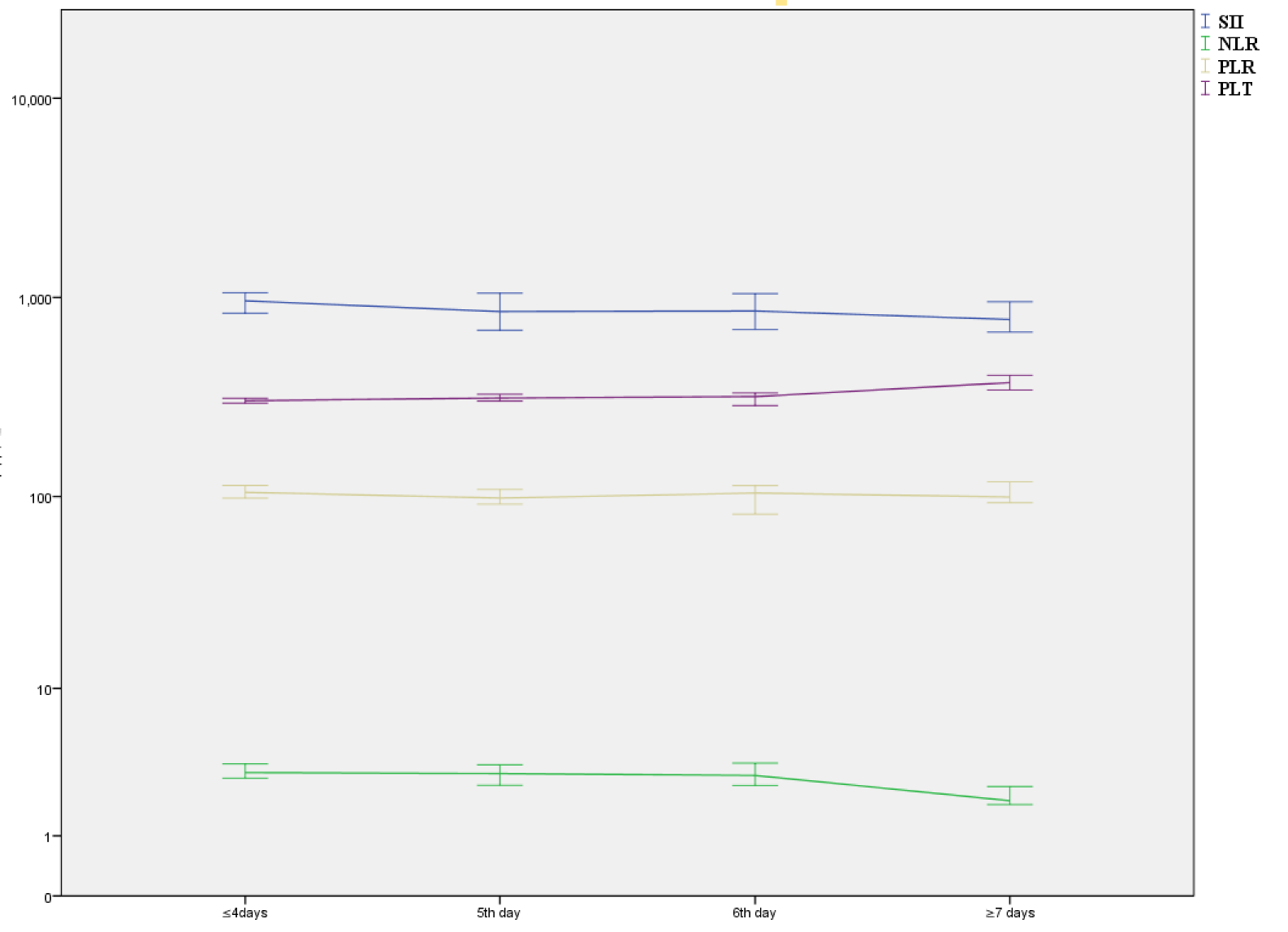


**The dynamic variability of PLT, NLR, SII, and PLR levels according to fever days.**
